# Acceleration without Disruption: DFT Software as a Service

**DOI:** 10.1021/acs.jctc.4c00940

**Published:** 2024-12-11

**Authors:** Fusong Ju, Xinran Wei, Lin Huang, Andrew J. Jenkins, Leo Xia, Jia Zhang, Jianwei Zhu, Han Yang, Bin Shao, Peggy Dai, David B. Williams-Young, Ashwin Mayya, Zahra Hooshmand, Alexandra Efimovskaya, Nathan A. Baker, Matthias Troyer, Hongbin Liu

**Affiliations:** † Microsoft Research AI for Science, Beijing 100080, China; ‡ Microsoft Azure Quantum, Redmond, Washington 98052, United States; § Microsoft Research AI for Science, Shanghai 200232, China

## Abstract

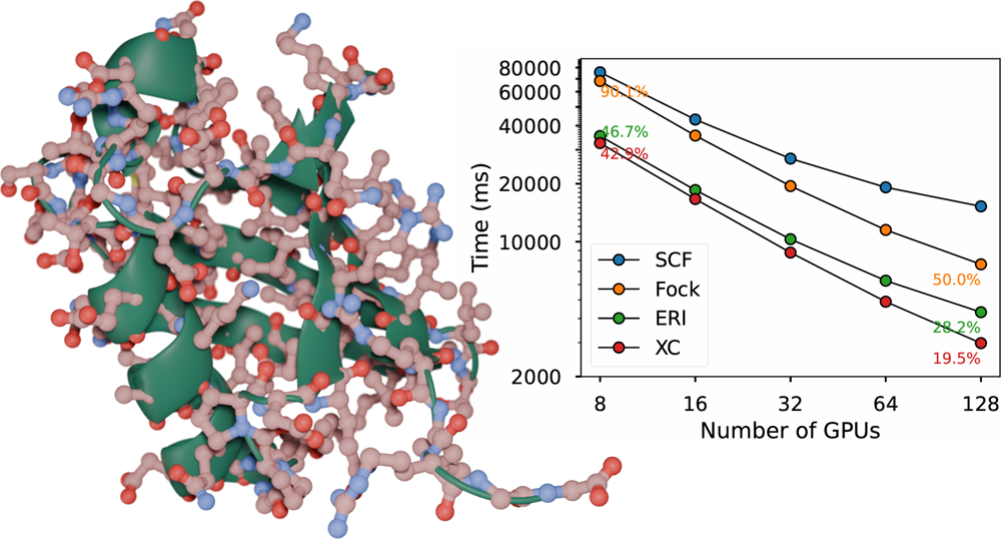

Density functional theory (DFT) has been a cornerstone in computational chemistry, physics, and materials science for decades, benefiting from advancements in computational power and theoretical methods. This paper introduces a novel, cloud-native application, Accelerated DFT, which offers an order of magnitude acceleration in DFT simulations. By integrating state-of-the-art cloud infrastructure and redesigning algorithms for graphic processing units (GPUs), Accelerated DFT achieves high-speed calculations without sacrificing accuracy. It provides a user-friendly and scalable solution for the increasing demands of DFT calculations in scientific communities. The implementation details, examples, and benchmark results illustrate how Accelerated DFT can significantly expedite scientific discovery across various domains.

## Introduction

Density functional theory (DFT) is a versatile and widely used computational method to study the electronic structure of chemical systems. From OLEDs^1,2^ to drug discovery,^3−7^ DFT has been crucial for understanding the underlying mechanisms that give rise to specific properties in materials, and for designing new functional materials. Its widespread applicability across various scientific domains has made it one of the most cited theories in the history of physics.^8^ Over the years, the accuracy of DFT calculations has been improved by development of various exchange-correlation functionals^9−24^ and the theory has been extended to study excited states and electronic transitions using time-dependent density functional theory (TDDFT).^25−28^

The increased utility of DFT is due not only to the development of the theory itself, but also to the dynamic interplay between advancements in programming languages, parallelization techniques, and more significantly, the increasing computational capabilities of hardware over the past five decades. The advancement in computer hardware, especially graphic processing unit (GPUs), has made it possible to reach higher speeds of DFT calculations through hardware-oriented implementation and optimization of DFT codes.^29−43^ These developments have dramatically altered the landscape of computational sciences, enabling more complex and large-scale simulations than were previously attainable. This increased capability was achieved without implementing approximations such as density fitting^44,45^ and other reduced-scaling techniques,^35,36,46,47^ which require trade-offs between accuracy and speed.

Given the increasing complexity of chemistry problems and the demand for rapid digital discovery of new chemicals, there is a critical need for more efficient DFT codes that can perform high-speed calculations with high accuracy. This demand is exemplified by the use of machine learning (ML) models for discovery of new molecules and chemicals, which has shown great potential in accelerating the discovery process by leveraging large-scale DFT data sets.^48−53^ These models can rapidly predict chemical properties, significantly speeding up high-throughput screening and exploration of chemical space for the discovery of new molecules.^54^ The size and accuracy of the data sets used for training these models have a significant impact on performance, further highlighting the need for DFT codes that can provide highly accurate data with high efficiency.

Here, we present Accelerated DFT, a computational chemistry service for electronic structure calculations of molecules. It offers a cloud-native, GPU-first approach for the implementation of DFT. Due to this approach, Accelerated DFT is able to harness the full computational power offered by contemporary cloud infrastructure and current GPU technologies, achieving an order of magnitude speedup in DFT simulations when compared to other programs using the same GPU or similar CPU cloud resources.

In this paper, we first provide an overview of the foundational design philosophy behind the key Accelerated DFT algorithms. We then detail the redesign and implementation of key components in a conventional DFT workflow. Next, we examine the user experience, highlighting the ease of use of the code. We present benchmark results comparing the code’s performance against other computational chemistry codes along with its scalability. Finally, we discuss how this software will accelerate scientific discovery.

### Cloud-Native Architecture Design

Our work on hardware-oriented GPU acceleration was inspired by the prior work of others in this space such as TeraChem,^29−32^ QUICK,^33,34,55^ GAMESS,^42,43^ EXESS^39−41^ and FermiONs++,^35−37^ as well as libraries such as BrianQC,^56^ LibintX,^38,57,58^ and GauXC.^38,59^ Over the past decade, rapid advancements in GPU technology have made these processors even more suitable for quantum chemistry calculations due to increased computational power, improved capabilities in double-precision calculations, enhanced parallel-processing capabilities, better memory management, and more mature software frameworks. The latest GPUs are often more readily available in the cloud due to their limited availability. Furthermore, cloud computing is becoming increasingly powerful for scientific workloads due to advances in network technology and high-performance computing architectures. For example, some Azure Virtual Machines (VMs) use InfiniBand technology, which provides high-performance, low-latency communication between nodes at scale, a capability that was once exclusive to selected facilities.^60^ These advantages have led us to adopt a cloud-native approach in the design of Accelerated DFT. As a result, Accelerated DFT will be offered as a service through an application programming interface (API) using specialized cloud hardware infrastructure, particularly InfiniBand and Remote Direct Memory Access (RDMA), to ensure optimal performance.

### GPU-First Algorithm Re-Design

To harness the full potential of GPU computing power, we deconstructed the DFT calculations process, and followed up with a comprehensive redesign and implementation. Below, we examine the DFT calculations workflow, and then delve into the intricacies of each critical component.

#### DFT Calculation Flow

The DFT computational process is characterized by a sequence of steps designed to ascertain the electronic structure of a given system. Here is a detailed breakdown of these steps:

- *Initialization of electron density*. The process begins by positing an initial estimate for the electron density, ρ(**r**), which is often derived from superposition of atomic densities or a previous calculation.^61,62^
- *Formation of the Kohn–Sham Fock matrix*. Based on the initial estimate, the Kohn–Sham Fock matrix can be constructed as
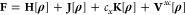
1where **H**, **J**, **K** and **V**^*xc*^ are the electron kinetic and external potential, Coulomb, exact exchange, and exchange-correlation (EXC) potential matrices, respectively.
- *Solving the Kohn–Sham equations*. The Kohn–Sham Fock matrix is diagonalized to obtain the eigenvalues and eigenfunctions, known as Kohn–Sham orbitals. This step is performed iteratively, as the orbitals depend on the electron density, which is concurrently being updated.
- *Density update and self-consistency loop*. The electron density ρ(*r*) is updated based on the occupied Kohn–Sham orbitals. The updated density is then used to recalculate the Fock matrix, and this loop continues until self-consistency is achieved—that is, until the input and output densities are in agreement within a specified tolerance. Direct Inversion in the Iterative Subspace (DIIS) algorithm^63,64^ is usually used to update the density and accelerate the convergence.

Accordingly, it can be deduced that the computational speed of DFT depends on the speed of each iteration and the total number of iterations needed to reach convergence. A better initial guess and more efficient update algorithms, such as DIIS, are helpful in controlling the number of iterations.^65,66^ Many mature methods already exist in this regard and are adopted by most DFT software.^67,68^

Further acceleration can be realized by completing each iteration faster. In each iteration, the most time-consuming part is the construction of the Fock matrix. For each element in *F*_μν_, the computational complexity can be calculated by following formula:
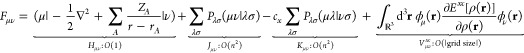
2where **P** is the density matrix, ϕ is the basis function, and *n* is the number of basis functions. For large systems, constructing the **J** and **K** matrices represents the largest computational cost, while for smaller systems, the **V**^*xc*^ term dominates due to its larger prefactor. In practice, the **J** and **K** matrices can be calculated by the Electron Repulsion Integrals (ERIs), which is an analytical integral, and the **V**^*xc*^ matrix is calculated by a numerically via quadrature. We refer to the latter integral as the Exchange-Correlation Energy (EXC) calculation. In addition to the construction of the Fock matrix, its diagonalization has an *O*(*n*^3^) complexity. The execution of the DIIS update algorithm also requires a considerable number of matrix operations.

The primary optimization effort in Accelerated DFT has been dedicated to the development of highly efficient algorithms for (1) the evaluation and direct contraction of the ERIs (and their derivatives) to form **J**, **K** and their contributions to analytical energy gradients, (2) the evaluation of the numerical EXC integral and its gradient, and (3) implementations of the Fock diagonalization and DIIS SCF acceleration on GPU architectures. We consider each of these optimizations in the following sections.

#### Key Components Redesign and Implementation

##### Electron Repulsion Integrals

In eq 2, during the construction of **J** and **K** matrices, the term (μλ|νσ) denotes the electron repulsion integral (ERI):
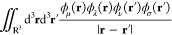
3For a Gaussian-Type Orbital (GTO) basis set, this integration can be efficiently computed by analytical integral methods, such as the Head-Gordon-Pople (HGP)^69^ and Rys quadrature^70^ algorithms. However, these integration methods pose significant challenges for implementation on GPUs. First, integral screening methods that work well on CPUs, such as those based on the Cauchy-Schwarz inequality,^61,71^ are hard to implement efficiently on GPUs due to reduced parallel efficiency resulting from varying computational work post screening. Second, these methods are based on recursive implementations, which have very complex computational graph dependencies. Moreover, for different angular momentum combinations, there is significant variability in the optimal computational graph paths. These calculations are vastly different from regular matrix computations for which GPUs have been designed, making them highly unfavorable for parallel execution on GPU architectures.

**Figure 1:**
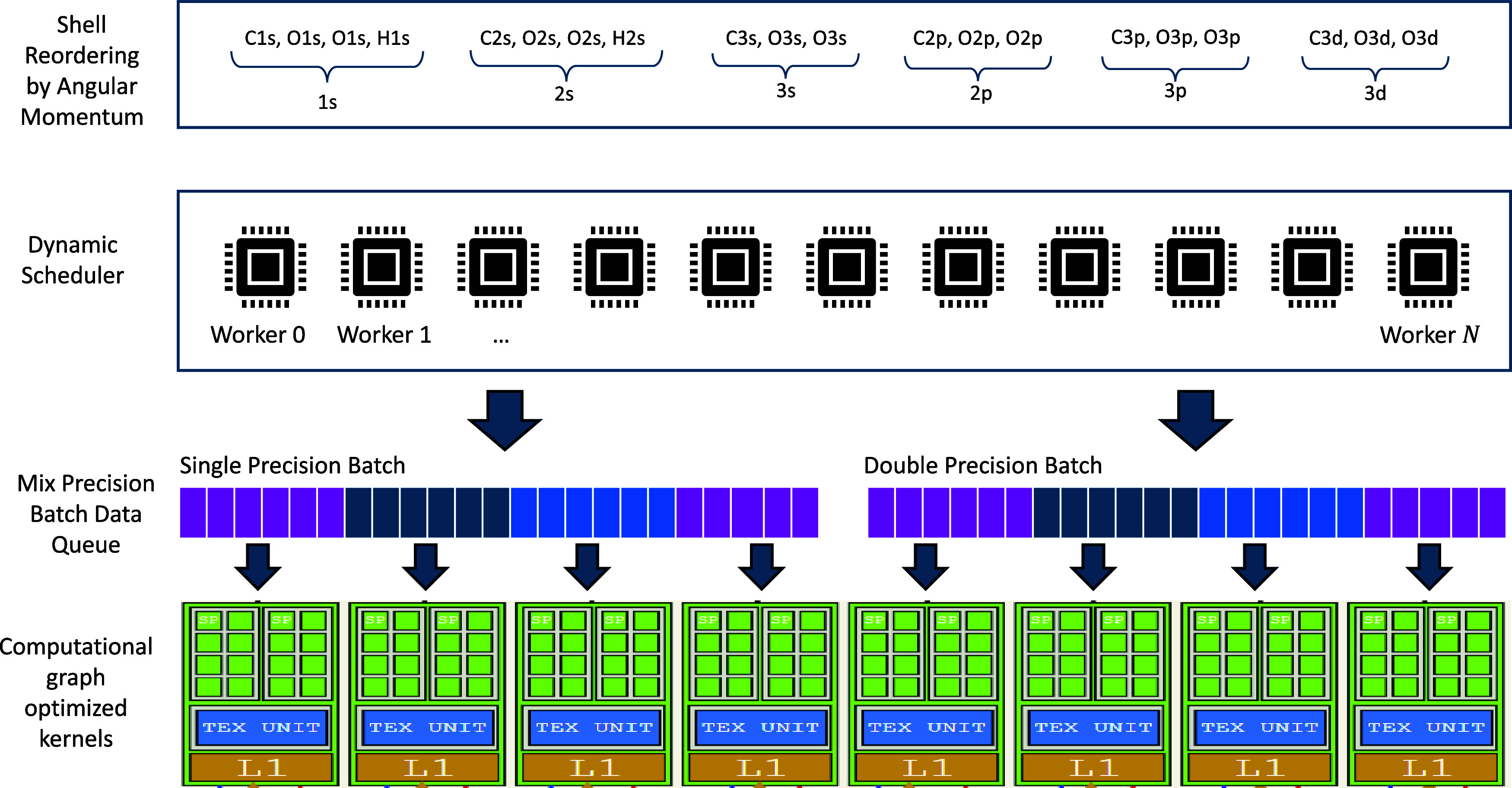
Electronic repulsive integral workflow as implemented in the Accelerated DFT code. The workflow starts by reordering the angular momentum and grouping the angular momentum pairings. The pairs are then sent to multiple workers for Cauchy-Schwarz screening. Based on the accuracy, the filtered batches are assigned to mixed precision batch queues. Finally, the batches from each queue are processed by an optimized GPU kernel.

In response to these challenges, we have pursued the following optimizations for the computation of ERIs on modern GPU architectures:

- *Dynamic scheduling of batch data with GPU-friendly angular momentum reordering*. Given that different combinations of angular momentum have completely different recursive computational graphs, we first need to reorder the angular momentum. For this reason, data is prepared in batches based on the combinations of angular momentum. In addition, basis contractions are taken into account in the reordering process, i.e., the scheduler treats multiple contractions with the same angular momentum as different shells. For example, all integrals with the angular momentum combination of (*pp*|*dd*) are grouped together, while those with (*dd*|*dd*) are in another group. In each group, multiple CPU workers perform Cauchy-Schwarz filtering on each integral. The integrals selected for calculations are placed in the batch data to be sent to the GPU. To mitigate the overhead of transferring batch data from CPU memory to GPUs, we utilize multiqueue processing and leverage the asynchronous computing capabilities of the GPU. Specifically, for each GPU, we assign multiple CPU workers with multiple GPU streams. After processing a batch of data, each worker submits it to a stream of GPU for calculation. Then, instead of waiting for the GPU calculation to complete, the worker starts processing the next batch. The GPU automatically processes the computation tasks in a first-in-first-out manner within each stream. By employing multiple workers and streams concurrently on each GPU, we significantly reduce the data processing overhead and achieve GPU utilization rates exceeding 99%.
- *GPU kernel code optimized for each type of angular momentum*. Each type of angular momentum combination has a significantly different recursive computational graph, and the GPU is not suitable for handling a large number of branch choices. Further, optimal computational pathways for low angular momentum ERIs such as (*ss*|*ss*) and (*ps*|*ps*) are often different from high angular momentum ERIs due to differences in resource utilization (e.g., registers, shared memory, etc.). Thus, it is necessary to develop bespoke solutions for the various edge cases to ensure maximal performance. To address this challenge, we have developed an offline optimization strategy that transverses the HGP graph to determine optimal pathways for each angular momentum combination, thereby maximizing the operating efficiency of each integral kernel on the GPU.
- *A mixed-precision strategy with almost no loss of accuracy*. The efficiency of calculations of different precision on a GPU varies significantly when compared to a CPU. To fully exploit the performance of the GPU, we developed a mixed single and double precision strategy. Specifically, we implemented both double precision and single precision versions for RYS and HGP algorithms, and a job dispatcher to decide how to “mix” them. The job dispatcher estimates the upper bound of a shell quartet using the Cauchy-Schwarz inequality, then selects the single precision version if it is enough to obtain the required precision. For example, consider a shell quartet of *A*, *B*, *C*, *D* with the estimated upper bound of *S*(*AB*|*CD*).
  1. If *S*(*AB*|*CD*) < TOLERANCE (10^–12^ by default) → There will not be dispatch to any algorithm;
  2. If TOLERANCE < *S*(*AB*|*CD*) < TOLERANCE ∗ 10^5^ → The single precision version is enough to obtain required precision;
  3. If *S*(*AB*|*CD*) > TOLERANCE ∗ 10^5^ → The double precision version is necessary.
  After Cauchy-Schwarz filtering, each group of integrals is sent to either a single precision batch or a double precision batch according to the estimated integral precision requirement. When enough data is accumulated in a batch, the corresponding single or double precision GPU kernel is called to perform the integral calculation in parallel. To match the GPU’s high computational speed, substantial engineering optimizations have been made to the scheduler to improve the efficiency of CPU worker data preparation. The overall workflow is depicted in Figure 1.

##### Exchange-Correlation Energy Integral (EXC)

**Figure 2:**
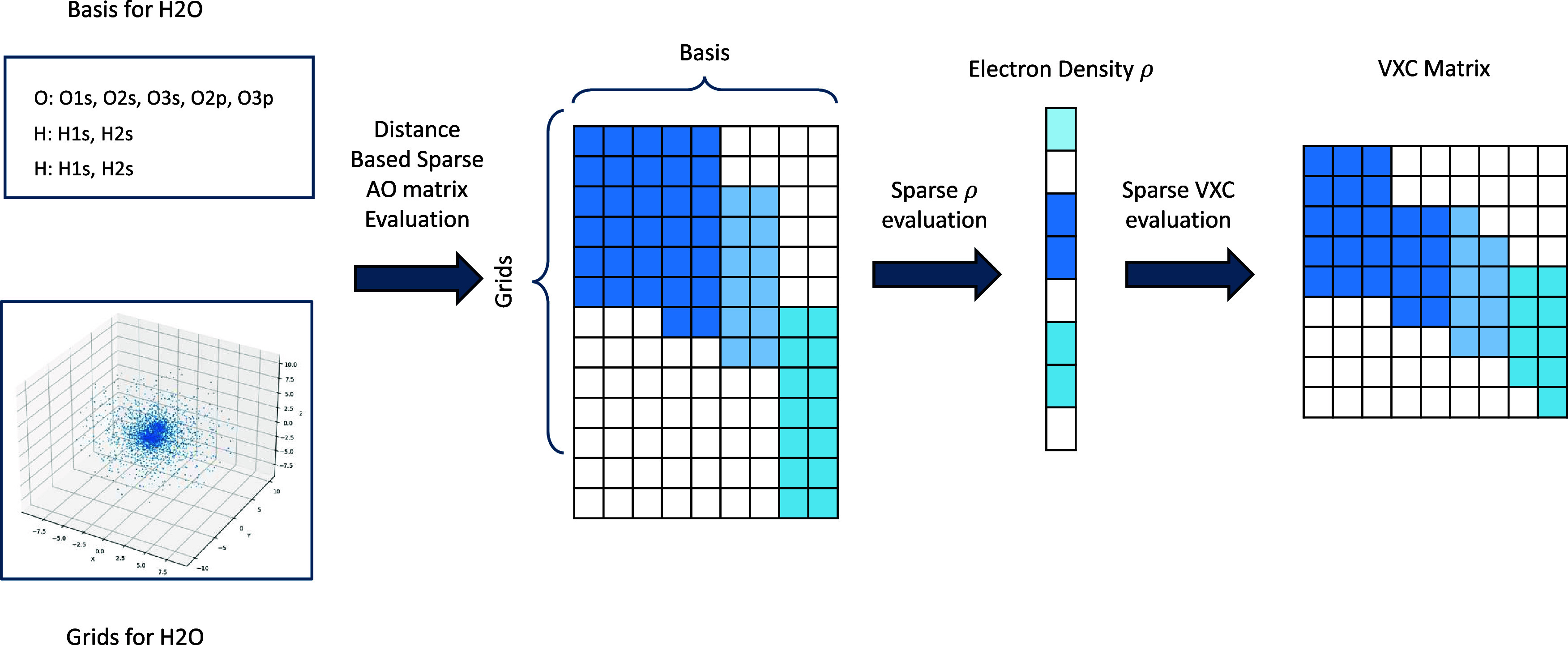
Exchange-correlation energy integral calculation workflow as implemented in Accelerated DFT for H_2_O as an example. For all the basis functions, the sparsity of the AO matrix on the grid points is constructed based on distance from atomic center. Using the obtained sparse AO matrix, the sparse ρ and **V**^*xc*^ computation modules are employed to yield the final output.

The calculations of EXC integral are very different from those of ERI. Due to the numerous types and complex forms of exchange-correlation functionals, EXC can usually only be calculated by numerical integration. Compared to ERI, numerical integration is relatively convenient to be implemented on a GPU because the calculations are regular, i.e., the same calculation is performed on each grid point, and then the grid points are summed. However, due to the exponential decay characteristics of GTO basis functions, it is possible to ignore the grid points that are far from the atomic center, since the integrated EXC values on those points are small. Taking advantage of this sparsity can dramatically impact the speed of the calculations. However, this sparsity creates challenges for GPU calculations. Although there are some sparse matrix optimization tools, their requirements for sparsity are high, and it is difficult to adapt to the calculation characteristics of EXC. In response to this challenge, we have designed a new sparse optimization algorithm for EXC:

- *Distance-based sparse atomic orbital (AO) matrix evaluation*. Considering the characteristics of the basis function, we set a precision threshold, and for each basis function, we calculate the maximum grid point distance with a value higher than this threshold. For grid points that are farther than this distance, the function is filtered from the data. This saves a considerable amount of computation. To leverage this sparsity, we have optimized the GPU kernels specifically for the computation of the AO matrix.
- *Adaptive sparse block matrix multiplication for* ρ *and***V**^xc^*evaluation*. In order to achieve efficiency gains, it is imperative that we fully exploit the sparsity of the AO matrix during the subsequent computations of the ρ and **V**^*xc*^ matrices. These computations are heavily reliant on matrix operations. Conventional acceleration techniques for sparse matrices typically require a high degree of sparsity and are not suitable for the weakly sparse nature of the ρ and **V**^*xc*^ matrices. Conventional techniques also fail to take advantage of symmetry, thereby impacting efficiency. To address this challenge, we have redesigned a novel matrix computation kernel within the Accelerated DFT framework, to thoroughly capitalize on the specific block-sparsity of the ρ and **V**^*xc*^ matrices. We use a fixed block size of 32 × 32 for our block-sparse strategy in matrix–matrix multiplication during the EXC calculations. The block is considered as zero if all of its 32 × 32 values are less than ϵ (10^–12^ by default). Zero blocks are not stored, while all values in nonzero blocks, including zero value, are stored in a dense manner. Conventional kernels, like cuBLAS, cannot be applied on such a data format directly. We implemented a modified version of tiled matrix–matrix multiplication algorithm for this block-sparse format. The overall workflow for EXC calculation is depicted in Figure 2.

##### SCF Iteration

As analyzed earlier, the construction of the Fock matrix consumes a lot of time, and once ERI and EXC calculations are moved to the GPU, the diagonalization of the Fock matrix and the DIIS algorithm create new bottlenecks if left in CPU. As a result, the diagonalization and DIIS operations are also implemented in GPU. For Fock diagonalization, we use cuSOLVER^72^ for solving, and cuBLAS^73^ to complete various matrix operations in DIIS.

InfiniBand and RDMA have been used ubiquitously in most of the code implementations. Even though DFT parallelization only involves broadcasting several *N*^2^ matrices, and conceptually does not rely heavily on a high-speed network, for large-scale DFT calculations, the exchange of matrices takes a non-negligible portion of the total execution time. InfiniBand ensures performance efficiency in those cases. As another example, the dynamic scheduling in ERI and EXC calculations requires frequent updates to a global task counter. We maintain a global shared counter on the rank#0 process, which needs to be accessed and updated by each worker node. This counter is updated hundreds of thousands of times during a single SCF step in the ERI calculation. The frequent update to the global counter makes the process highly sensitive to data communication latency. RDMA allows us to perform these updates with minimal delay, since it provides the low-latency memory access that is crucial for maintaining the efficiency of our dynamic scheduling algorithm. RDMA uses InfiniBand, which offers both high bandwidth and low-latency communication. This reduces the time spent on data transfer. Additionally, RDMA operations bypass the remote CPU and OS kernel, which leads to lower CPU overhead. This is particularly beneficial in our distributed CPU-GPU heterogeneous computing environment. By leveraging RDMA we can achieve significant performance improvements in our DFT framework. The reduced latency and CPU overhead directly enhance the efficiency of our dynamic scheduling, which leads to faster and more scalable computations.

### Minimized Redundancy

Throughout the history of DFT code development, there has been a tendency to create comprehensive packages that aim to meet the diverse needs of researchers using the programs. This is because the application of DFT is incredibly varied, with different users focusing on distinct aspects, such as energy, forces, geometry, and other properties. Attempting to encompass all of these facets would result in a significantly larger code base than one designed only for solving the Kohn–Sham equations. This point is illustrated by considering some popular open-source DFT programs. For instance, CP2K v8.2^74^ has more than 900,000 lines of FORTRAN code, and Psi4 v1.3.2^75^ has nearly a half million lines of C++ code. The diversity of ways that users consume DFT has resulted in duplicated efforts in utility implementations. For example, almost every DFT code has its own implementation for orbital localization and population analysis, among other tasks.

To avoid redundancy when developing a new DFT program, it is best practice to evaluate which parts of the design and build require new code, and what features and functions could be built by leveraging existing tools. Our approach in developing a new DFT code is to focus on aspects that significantly enhance performance and provide users with new features and value, rather than attempting to address every possible application or functionality. Thus, Accelerated DFT leverages mature, open-source software libraries in the build of nonperformance-critical features.

We have adopted open-source software for the following aspects:

1. *Exchange-correlation functional*. The support of various exchange-correlation functionals is the key feature of any DFT code. LibXC^76^ is a mature package that is used in many DFT codes and we employ it to generate the functional form, which is a compute-trivial task. It also provides agile support for new functionals released yearly.
2. *One-electron integrals (with pseudopotential)*. One-electron integrals contribute to the dominant terms in the Fock matrices, however, their compute complexity is low. We use Libint^77^ to compute the standard one-electron integrals and Libecpint^78^ to compute one-electron pseudopotential integrals.
3. *Dispersion*. van der Waals dispersion corrections usually have a very simple analytical form and a very low intensity of compute. These corrections play a critical role in accurately predicting the electronic structure of molecules that include a noncovalent bond. We used simple-dft3 to introduce the D3 correction.^79^
4. *Solvation models*. Solvent can drastically change the electronic structures of molecules. Implicit solvation models like polarizable continuum model (PCM) usually strike a good balance between the cost and accuracy of describing realistic environments. For systems containing less than hundreds of atoms, constructing the PCM cavity and solving Poisson’s equation does not affect performance considerably and thus has not been a practical concern in our present studies. However, for very large systems, these costs can be immense, and scalable solutions should be sought.^80−82^ We currently use PCMSolver^83^ to introduce solvation into the Accelerated DFT code implementation; other, scalable solutions may be required in the future.
5. *Geometry optimizer*. We use geomeTRIC^84^ as an optimizer to execute geometry optimization, one of the most commonly performed tasks in any DFT code.
6. *Input/Output schema*. QCElemental^85^ and QCSchema^86^ were used to standardize and serialize the input and results.
7. *Extensibility*. We use QCEngine’s ProgramHarness^87^ to abstract the execution of the DFT code. The program also ensures the future extensibility of the code to seamlessly integrate modified, improved theories for DFT calculations of electronic structure.
8. *Various other utilities*. One of the great challenges in quantum chemistry is the diverging data format of different codes, which makes integration with other tools difficult. To overcome this, our strategy has been to align the data format of molecular orbitals (MOs), density matrices, etc. with PySCF.^88^ Therefore, the binary output written by Accelerated DFT can be directly consumed and read in PySCF. Another advantage of this strategy is the generation of additional properties, such as the g-tensor of IR spectra, by calling the corresponding PySCF utilities to recreate the SCF object once the binaries are loaded.

## User Experience

In recent years, cloud computing has shaped a new landscape for HPC. In response, we are introducing a new paradigm in user experience and operational methodology. Below, we discuss key features of the user experience.

From a user perspective, other than the performance of a DFT code, the most important feature is its ease of use. In the contemporary landscape of computational chemistry, running DFT calculations is a process that typically begins with the installation or compilation of specialized software. Due to the computationally intensive nature of DFT, the performance of these programs often hinges on how well they are optimized for the specific hardware on which they run. Scientists typically compile the software directly on their high-performance computing systems to ensure maximum efficiency. This process involves configuring the software to leverage the specific architecture and capabilities of the processors, memory, networks, and sometimes even specialized accelerators such as GPUs.

Once the software is properly installed and optimized, users initiate DFT calculations through command-line interfaces (CLI). The preparation of the input files is a critical step in this process. These files, typically in a text format, must adhere to program-specific syntax and structure. The files contain detailed information about the system of study, including atomic coordinates, basis sets, functionals, and other computational parameters. These files serve as the API of the DFT program. Such design has its origin in historical practices, but it creates a tight restriction on the collocation of user and compute environments.

### API

The foundation of a cloud service, as well as the starting point of the user experience, is the API. The purpose of the API is to distill all operations of the HPC environment to the single element of compute, i.e., only the calculation itself is performed on a standard HPC platform and all the extraneous tasks such as preparation of the calculation are executed on any routine computing resource. For example, a common format of a cloud API is the “Representational State Transfer Application Programming Interface” (RESTAPI),^89^ which is a set of rules that allows different computer programs to communicate with each other over the Internet. This technology enables apps and Web sites to seamlessly access and share information.

### Input and Output

The first step when using the RESTAPI^89^ is to decide which data will be exchanged during the communication, i.e., the data schema. Here, the data input is defined in a formal but readable syntax so users can focus on scientific content rather than figuring out any program-specific syntax. In modern cloud-app development, JSON is a popular input and output (I/O) data format.^90^ One advantage of the program is the prevalence of the JSON-based data schema, QCSchema,^86^ advocated by MolSSI to be the I/O standard for quantum chemistry applications. QCSchema has already been reviewed and adapted in the quantum chemistry software community, and is ideal for specifying the I/O parameters for this API application.

We have made two modifications to the pure QCSchema-based I/O. First, we acknowledge the likely persistent demand of running DFT calculations on a standard molecular structure format like xyz. Therefore, the Accelerated DFT service can start the calculations either with a single QCSchema input or with a legacy structure file like xyz, supplemented by a JSON input in the RESTAPI request that specifies the parameters for the DFT calculations. Second, output formats in traditional DFT are not only text, but binary as well (e.g., checkpoint files). In the context of a cloud service and RESTAPI, it is not efficient to always stream binary outputs, which typically range from dozens of megabytes to gigabytes, from the service to the client. To solve this issue, Accelerated DFT service integrates with the user’s Azure Blob Storage account, which is an object store solution in the cloud. The binary output is stored within a container in the Blob Storage Account.^91^ The user can access the binary files using uniform resource identifiers (URIs) listed in the RESTAPI response.

### Job Interaction

Upon defining the API and the I/O for Accelerated DFT service, a crucial aspect is determining the methods through which users can interact with the service. In consideration of traditional practices in DFT usage and the growing demand for automation and scalability, we have implemented a Command Line Interface (CLI) using the Azure CLI. This decision honors the conventional approach familiar to many in the scientific community while also catering to the needs of large-scale computational tasks and automated workflows.

Furthermore, recognizing the importance of seamless integration with other computational tools and workflows, we have developed a Python Software Development Kit (SDK). This SDK is designed to facilitate easy integration of our DFT service into a wide range of computational chemistry environments. It allows users to programmatically access DFT calculations, making it simpler to incorporate these tasks into larger, more complex workflows. The Python SDK is particularly advantageous for users who seek to combine DFT calculations with other computational tasks, such as data analysis, machine learning, or other postprocessing activities.

The CLI and Python SDK make it convenient for the user to interact with the RESTAPI as well as their Azure Blob Storage Account by providing abstractions for uploading input, submitting DFT calculation requests, and downloading output. For example, the code in line 23 of Listing 1 will interact with the RESTAPI and the Azure Blob Storage account to download the output of the DFT calculation.

In traditional DFT codes, job management is usually not in the scope of the software. Users rely on external job schedulers or managers like SLURM or PBS to perform tasks such as scheduling and querying jobs for status. However, the DFT cloud service, presented here, has integrated job management functionality. Jobs can be queried through a web portal, the CLI, or Python SDK. List 1 shows an example of how users could submit DFT calculations using the Python SDK and integrate with PySCF for convenient result analysis.
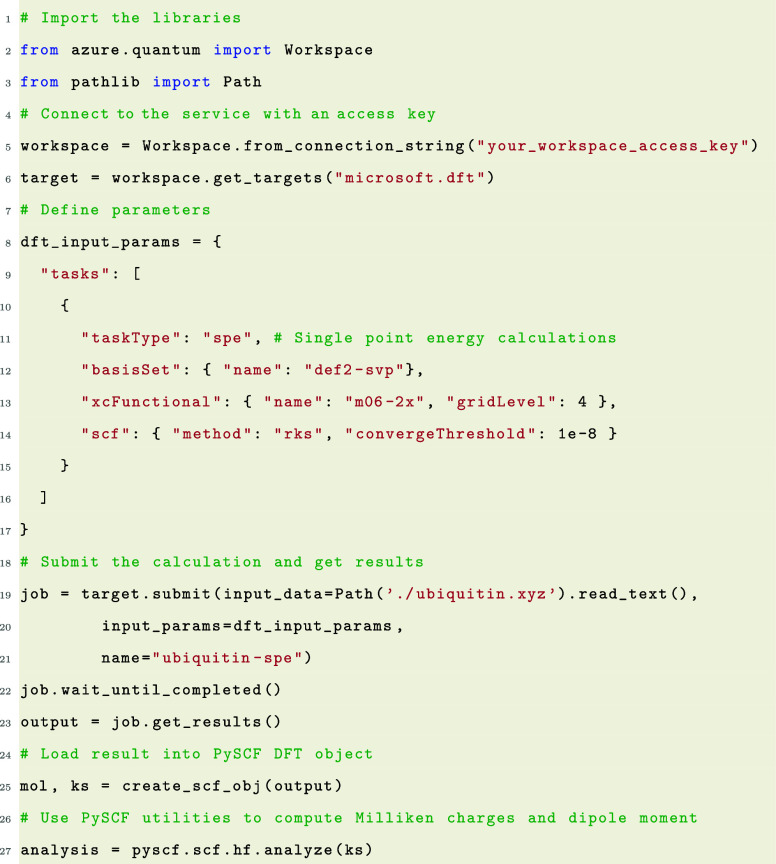
Listing 1: Example of submitting a synchronous DFT calculations using Accelerated DFT service through Python Software Development Kit. As the data format of Accelerated DFT has already been aligned with PySCF, the Accelerated DFT results stored in the QCSchema format can be load directly as a PySCF object. PySCF functions can be utilized directly for result analysis. The Python SDK and CLI abstract the RESTAPI and Azure blob storage interactions for the user. The “output = job.get results()” line in the above sample downloads the data from the Azure blob storage.

Lines 8–21 in List 1 could be further compressed into the code snippet in List 2 if the input file format is QCSchema.
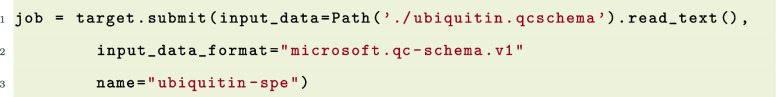
Listing 2: Example of submitting a DFT calculation using QCSchema to Accelerated DFT service through Python Software Development Kit.

## Tasks

A common question for any new DFT code is, “What can users do with it?” In the design of Accelerated DFT, a key user-experience decision was made to streamline the range of tasks available to users, in contrast to the extensive options provided by traditional DFT packages. Specifically, Accelerated DFT limits its API to accept only five specific tasks: single-point energies (spe), single-point forces (spf), full analytical Hessian calculations (fh), geometry optimizations (go), and Born–Oppenheimer molecular dynamics (BOMD).^92^ The rationale behind this focused approach is rooted in the fundamental nature of DFT calculations and the evolving needs of the scientific community.

Single point energies, single point forces and full Hessians constitute the core and fundamental properties to be evaluated in DFT calculations. These basic calculations lay the groundwork from which a multitude of properties can be derived, such as electron density, population analysis, g-tensors, hyperfine coupling tensors, NMR spectra, IR spectra, and thermochemical properties. Tasks like NMR calculations are identified as separate functionalities in almost all traditional DFT codes; however, in Accelerated DFT, they are viewed as derivative tasks stemming from the three core calculations. This configuration not only simplifies the API but also streamlines the computational process. As previously noted, the primary optimization effort in Accelerated DFT has been the optimization of components required for the efficient evaluation of spe and spf (integral and analytical integral gradients) on GPU architectures. As such, all supported methods, including pseudopotential calculations as well as PCM and D3 corrections to the DFT energy, admit analytical spf implementations. Initial efforts have been made to integrate these components into the fh evaluation, but vast portions of this workflow remain on the CPU. Pursuance of fully accelerated fh calculations in Accelerated DFT will be the subject of future work by the authors. However, we note that seminumerical evaluation of the energy Hessian (via numerical differentiation of the spf) remains a fully GPU-accelerated workflow. Further, due to limitations resulting from our use of third party libraries to provide agile feature support to users, fh tasks are currently not compatible with PCM or D3 corrections to the DFT energy.

Geometry optimization is essentially a serial collection of single-point force calculations. However, it is included as a standalone task due to its widespread use across the entire field of computational chemistry. It remains one of the most commonly executed jobs, necessitating its presence as a primary function in the service. We note that “go” is supported in Accelerated DFT for all methods that support analytical gradients.

The inclusion of BOMD was a forward-looking strategy. Historically, BOMD has seen limited use due to its prohibitively expensive computational cost. However, with advancements in DFT efficiency and the growing interest in using BOMD as a method for sampling potential energy surfaces and chemical spaces, its relevance has increased. This is particularly pertinent in the context of generating data for machine learning potentials and AI applications in chemistry. By supporting BOMD as an individual task, Accelerated DFT aligns itself with these emerging trends and supports the broader scientific community’s efforts in these areas. Several examples of input files for different tasks are provided in SI. As with geometry optimizations, BOMD is supported for all methods in which analytical gradients are implemented.

## Benchmarks

In this section, we present comprehensive benchmarks for the performance of Accelerated DFT in comparison to other well-known codes and in its scalability. For the first case, we compiled a diverse set of molecules, carried out DFT calculations on a single node using Accelerated DFT, PySCF,^88^ GPU4PySCF,^93^ Psi4,^75^ and TURBOMOLE,^94^ and provided the accuracy and runtime of Accelerated DFT in comparison to the other codes. For the second case, the scaling performance of Accelerated DFT was analyzed with a focus on the parallel efficiency of the code. This allowed us to identify the optimal balance between time and computational cost, providing a reasonable time-to-solution while analyzing extremely large molecules.

### Comparative Performance Benchmarking: Accelerated DFT vs Other Codes

Benchmarking new DFT codes against established ones is crucial for evaluating their performance. In this regard, it is important to assess the performance of the codes on a diverse set of chemical structures, not only to evaluate the robustness of the codes, but also to detect their limitations in handling different chemical systems and to validate their reliability. While many benchmark data sets have been produced and adopted during decades of development of DFT, most of those data sets were created with the purpose of benchmarking the accuracy of new functional development, rather than testing the efficiency of one DFT code versus another.^23,95,96^

To evaluate the performance of Accelerated DFT in comparison to other available DFT codes, we created a diverse data set of molecular structures, in terms of both size and chemical composition. This data set comprised 329 molecules from the tmQM^97^ and PubChem databases.^98^ The tmQM data allowed access to molecules with catalytically active transition metals, whereas the selected PubChem data contained structures with diverse chemical compositions and large sizes. These molecules contained elements from rows 1–4 of the periodic table and a minimum of 100 atoms, each with at least four unique atom types. Of the 329 molecules, 110 contained transition metals (28 from the fourth row, 54 from the fifth row, and 28 from the sixth row), and two molecules contained the rare-earth element lanthanum.

For all calculations, the def2-tzvpp basis set^99^ was used, which contains polarization functions and provides for basis functions with high angular momentum. For example, 110 tests have up to *g* functions, 217 have up to *f* functions, and two have up to *d* functions. For the systems studied here, the choice of def2-tzvpp gives a range of 28–3,913 total basis functions. Moreover, def2-tzvpp implements effective core potential (ECP) in elements heavier than krypton to replace the core electrons. We used ECPs in 85 of the electronic structure calculations in the studied data set. The calculations were performed using both M06-2X,^100^ a hybrid meta-GGA exchange-correlation functional, and ωB97x,^101^ a range-separated hybrid GGA exchange-correlation functional, to evaluate the speedup of Accelerated DFT using different hybrid functionals. The XC numerical integrations were done on an integration grid equivalent or close to the level-4 grid setting in PySCF. A narrow convergence threshold was set to achieve high accuracy by using the ERI screening tolerance of 10^–12^ and SCF convergence threshold of 10^–8^. Analytical **J, K** formations were used exclusively throughout all calculations presented in this paper; no density fitting or any other numerical **J, K** builder was used. The design of these benchmark calculations means that existing software to be used for comparison must support the def2-tzvpp basis set and support ECP and high angular momentum basis functions (*f* and *g*) that arise due to this basis set. Additionally, the software must support full analytical evaluation of **J, K**. Within these limits, we compare Accelerated DFT to a set of codes optimized for CPUs: PySCF (v2.4),^88^ Psi4 (v1.9),^75^ and TURBOMOLE (v7.7),^94^ and the GPU-optimized software GPU4PySCF (v0.6.17).^93^

For all CPU-based codes, benchmark DFT calculations were carried out on the Microsoft Azure Quantum Elements platform, using nodes equipped with 120 AMD EPYC 7 V12 processor cores with 4 GB of RAM per CPU core. For Accelerated DFT, the calculations were performed on nodes with 4 NVIDIA A100 PCIe GPUs with 80GB memory each, and 96 AMD EPYC 7 V13 Milan cores. Since GPU4PySCF does not support multi-GPU computation, GPU4PySCF calculations were performed with 1 NVIDIA A100 PCIe GPUs with 80GB memory and 24 AMD EPYC 7 V13 Milan cores.

Within the SCF setting as described above, the convergence of the SCF cycle was obtained for all 329 molecules in the benchmark using Accelerated DFT. For the rest of the codes, the convergence was observed to be different as each of these codes has different default SCF optimization procedures, e.g., the number of maximum steps in the SCF cycle, the implementations of DIIS and incremental Fock algorithms, the Fermi level shifting, etc. The purpose of this benchmark is not to compare the convergence efficiency of the SCF cycle, but the efficiency of each step within the SCF cycle. Thus, we limited the comparison between the results of Accelerated DFT and other codes to the converged calculations within the same SCF convergence criteria as described above.

In comparing the results of Accelerated DFT with other codes used in the performance benchmark analysis, we deemed convergence as our first benchmark test since it demonstrates the robustness and stability of a computational code. For Accelerated DFT, all 329 calculations converged using both M06-2X and ωB97x functionals. However, for the other codes, the convergence was not achieved for all the molecules. For example, convergence was achieved in PySCF for 326 molecules with M06-2X (326 with ωB97x), in Psi4 for 323 (325), in TURBOMOLE for 307 (314), and in GPU4PySCF for 327 (327) with M06-2X (ωB97x) functionals, respectively. For the remainder of the discussion in this section, we compare the accuracy, runtime, and speedup between the converged results only.

In examining the total energies of individual molecules, the results from any two codes were considered consistent if the errors were below 0.001 hartree. Errors above this threshold, indicated a different local minimum of the KS wave function from two codes, resulting in fewer SCF iterations and shorter computation time in one code compared to another, which also made the speedup of one code over another incomparable. Setting this criterion for comparing the performance of Accelerated DFT against other codes led to exclusion of 13 (12) data points from PySCF using M06-2X (ωB97x), 10 (10) from Psi4, 21 (41) from TURBOMOLE, and 20 (13) from GPU4PySCF. The mean absolute error (MAE) was then calculated on the filtered results as presented in Table 1.

**Table 1:** Mean Absolute Error in the Total Energy of the Converged DFT Calculations Using M06-2X/ωB97x XC Functional^a^

	**Accelerated DFT**	**PySCF**	**Psi4**	**TURBOMOLE**
PySCF	0.027/0.016			
Psi4	0.088/0.026	0.086/0.023		
TURBOMOLE	0.526/0.200	0.530/0.203	0.510/0.188	
GPU4PySCF	0.027/0.016	0.000/0.000	0.086/0.024	0.530/0.203

a All numbers are in mHartrees.

When compared to Accelerated DFT, PySCF, GPU4PySCF and Psi4 show an MAE within the same order of magnitude, 10^–5^ Hartrees, with the smallest MAE values belonging to PySCF and GPU4PySCF due to their implementation of a grid identical to that of Accelerated DFT. TURBOMOLE, on the other hand, exhibits an MAE 1 order of magnitude larger due to differences in its grid implementation compared to Accelerated DFT. Errors with M06-2X are larger than with ωB97x; M06-2X may require a larger quadrature grid to converge to the grid limit.

Before we proceed with the analysis of the speedup of Accelerated DFT over other codes, it is essential to underscore two key aspects when evaluating relative speedup between codes. First, the computation time in DFT calculations relies heavily on the setup of the calculations, i.e., XC functional, grid level for evaluation of XC, convergence criteria, etc. This directly impacts the speedup of one code over another; the results from one benchmark with a specific calculation setup cannot be generalized to indicate the performance of a code in general. Second, any claims about higher efficiency of one code over another should be considered in the scope of performance on a large data set, i.e., in terms of “mean” speedup, because individual cases may represent outliers and deviate significantly from the mean performance of the code (See Figure S1).

Using the data set and the calculation setup as discussed at the beginning of this section, we calculated the mean speedup factor of Accelerated DFT over PySCF, Psi4, and TURBOMOLE, as summarized in Tables 2 and 3. To further highlight the efficiency of Accelerated DFT, pairwise mean speedup factors between the three other quantum chemistry codes in the benchmark are also reported in Table 2. Notably, while none of the pairwise comparisons among these established codes yielded a mean speedup factor greater than five, Accelerated DFT significantly outperformed all other codes in the benchmark. A schematic of distribution of data for each pairwise comparison is given in the Supporting Information (SI) section, Figures S1 and S2. It is noteworthy that individual cases in Accelerated DFT showed speedup factors several times larger than the mean value, as reported in the speedup range in Tables 2 and 3. A few examples reflecting the distributions of the speedup can be find in Figure S5. This reinforces the point made previously on assertions regarding the speedup of one code over another.

**Table 2:** Comparison of Speedup of Accelerated DFT and Leading Quantum Chemistry Software for M06-2X and ωB97x XC-Functionals on the Test Set^a^

**XC = M06-2X**				
		**PySCF**	**Psi4**	**TURBOMOLE**
Accelerated DFT	mean speedup	31.55	21.62	25.41
	speedup range	[12.93, 68.53]	[6.02, 50.37]	[3.09, 96.25]
	standard deviation	4.62	5.71	11.03
PySCF	mean speedup		0.68	0.84
	speedup range		[0.35, 1.40]	[0.24, 2.48]
	standard deviation		0.14	0.40
Psi4	mean speedup			1.37
	speedup range			[0.47, 4.52]
	standard deviation			0.89

**XC =** ω**B97x**				
		**PySCF**	**Psi4**	**TURBOMOLE**
Accelerated DFT	mean speedup	25.34	122.32	29.24
	speedup range	[9.00, 47.13]	[22.30, 191.26]	[4.92, 118.11]
	standard deviation	4.25	40.31	16.20
PySCF	mean speedup		4.70	1.20
	speedup range		[1.23, 6.65]	[0.55, 3.57]
	standard deviation		1.29	0.69
Psi4	mean speedup			0.34
	speedup range			[0.12, 1.43]
	standard deviation			0.30

a The mean, range, and standard deviation values are given as the speedup of the code on the left over the code on the right.

**Table 3:** Speedup Comparisons of Accelerated DFT and GPU4PySCF for M06-2X and ωB97x XC-Functionals on the Test Set^a^

		**GPU4PySCF 1 GPU**	
		**M06-2X**	ω**B97x**
Accelerated DFT 1 GPU	mean speedup	5.78	8.22
	speedup range	[1.59, 23.53]	[3.12, 28.42]
	standard deviation	2.49	4.07
Accelerated DFT 4 GPUs	mean speedup	17.38	25.61
	speedup range	[1.77, 60.24]	[3.37, 86.15]
	standard deviation	6.50	11.12

a The mean, range, and standard deviation values represent the speedup of the code on the left versus the code on the right. 1-GPU calculations were run on 1 A100 GPU and 24 CPUs. Accelerated DFT supports multi-GPU processing and the results of running the same calculations on a node with 4 A100 GPUs and 96 CPUs are also presented to show efficient parallelization and the advantage of Accelerated DFT in performing fast calculations.

Table 3 compares Accelerated DFT and GPU4PySCF for the 329 molecule test set. Accelerated DFT calculations were carried out with both 4 GPUs and 1 GPU for ease of comparison to GPU4PySCF, which supports only single-GPU calculations. For single-GPU calculations, Accelerated DFT shows a mean speedup of 5.78 for M06-2X, with an even greater speedup of 8.22 for ωB97X. In all cases, Accelerated DFT is faster, with the smallest speedup being 1.59 (3.12) for M06-2X (ωB97X). These numbers increase significantly for the 4-GPU calculations, demonstrating the efficient parallelization of Accelerated DFT.

We also compared performance of Accelerated DFT with GPU4PySCF calculations on water clusters of increasing size. Inputs and benchmark timing data for these calculations were taken from the GPU4PySCF Web site.^102^ Accelerated DFT and GPU4PySCF calculations were performed using 1 NVIDIA A100 GPU and 24 EPYC 7 V13 Milan CPU cores. Accelerated DFT supports multi-GPU computing so calculations with 4 NVIDIA A100 GPUs and 96 EPYC 7 V13 Milan CPU cores were also conducted. All timing data is presented in Table 4. We first note that GPU4PySCF calculations performed on the Azure platform were quicker than those provided by the GPU4PySCF authors–to calculate the speedup factors we use timings from the calculations on the Azure platform in order to provide a fair comparison. For small systems, i.e. up to 15 atoms, GPU4PySCF and Accelerated DFT show similar performance but, as the system size increases, Accelerated DFT significantly outperforms GPU4PySCF. The largest calculation using 8,201 basis functions shows a speedup factor of 3.94 on a single GPU and 12.64 on 4 GPUs. This speedup is less than that seen for the 329 molecule test set because the water clusters contain only light elements, with a single *f* basis function per oxygen, whereas the 329 molecule test set contained 110 molecules with *g* functions and 217 with *f* functions. This demonstrates the enhanced speed of Accelerated DFT when using high angular momentum basis functions, which are needed for accurate calculations.

**Table 4:** Performance Comparisons of GPU4PySCF and Accelerated DFT on a Series of Water Clusters of Increasing Sizes (NAtoms = Number of Atoms, NBasis = Number of Basis Functions)^a^

		**GPU4PySCF**				**Accelerated DFT**				
							**1 GPU**		**4 GPUs**	
**NAtoms**	**NBasis**	****official** time (s)**	****Azure** time (s)**	**cycles**		**cycles**	**time (s)**	**speedup**	**time (s)**	**speedup**
3	59	8.02	5.67	8		9	4.82	1.18	6.24	0.91
15	295	8.03	7.35	10		12	6.43	1.14	7.30	1.01
30	590	17.62	15.37	10		12	10.38	1.48	8.90	1.73
60	1180	81.65	74.30	12		14	32.51	2.29	14.94	4.97
96	1888	203.23	188.19	12		13	66.13	2.85	25.06	7.51
141	2773	454.99	421.96	12		13	139.29	3.03	46.86	9.01
228	4484	1482.62	1388.79	14		15	439.75	3.16	128.78	10.78
300	5900	2313.47	2190.85	12		15	637.49	3.44	209.17	10.47
417	8201	5401.69	5229.57	14		15	1327.83	3.94	413.82	12.64

a GPU4PySCF “Official times” are taken from the GPU4PySCF website,^102^ and “Azure Time” refers to the same GPU4PySCF calculations run on the Azure platform. The number of cycles required for convergence is also reported. All times are total compute time for the calculation. Calculation details: M06/def2-tzvpp basis set, 1 × 10^–9^ energy convergence threshold, integration grid = level 4. GPU4PySCF calculations were run with 1 A100 GPU and 24 CPUs (a quarter of the node). Results of Accelerated DFT with a single GPU are also provided for a clear comparison with GPU4PySCF. Accelerated DFT supports multi-GPU computing and the results of running the calculations on a node with 4 A100 GPUs and 96 CPUs are provided to show efficient parallelization in the code.

In summary, Accelerated DFT clearly exhibits robustness and efficiency. Its substantial speed, when measured against widely used quantum chemistry software programs, highlights its benefits in supporting high-throughput calculations and modeling complex molecular systems.

### Scaling Performance Benchmarking: Accelerated DFT on Multiple Nodes

To benchmark the scaling performance of Accelerated DFT, we studied ubiquitin, a large molecule comprising 1,231 atoms,^59^ on 1–16 nodes. The calculations were carried out on a cluster of Azure ND A100 v4 series virtual machines. Each node has 8 NVIDIA A100 40G GPUs and 96 AMD EPYC Rome CPU cores. GPU nodes are connected with 200 GB/s NVIDIA Mellanox HDR InfiniBand connections. All GPUs were configured with one MPI process per GPU.

The def2-svp basis set and the M06-2X^96^ exchange-correlation functional were used for the DFT calculations. All calculations were performed until a convergence of 10^–7^ in SCF and 10^–12^ in the ERI screening was achieved. For XC integral evaluation, PySCF grid level 4 was implemented, which has 60 radial and 434 angular points for first row elements, 90 radial and 590 angular points for second row elements, and 20,719,091 grid points in total after pruning. All calculations were converged with the exact same energy of −29, 772.5684767 hartree after 18 SCF iterations.

Table 5 presents the time to complete SCF calculations of ubiquitin on 8–128 GPUs. Additionally, it shows the time for completion of individual components within an SCF step to provide a detailed analysis of the scaling performance and parallel efficiency of Accelerated DFT.

**Table 5:** Time Required to Perform Energy Calculations (M06-2X/def2-svp) for Ubiquitin (1231 Atoms and 11,577 Spherical Basis Functions) on 8–128 A100 GPUs^a^

GPUs	total	SCF	Fock	ERI	XC	ERI_MPI (ms)	XC_MPI (ms)	Bcast_P (ms)
8	701.5	35.8	28.8	22.6	5.9	1152.9	581.4	423.0
16	480.1	24.0	17.0	12.9	3.7	1193.1	602.4	454.9
32	369.3	18.1	11.1	8.0	2.7	1254.5	622.2	492.4
64	319.2	15.4	8.3	5.7	2.3	1309.4	645.2	571.7
128	297.1	14.0	6.9	4.5	2.1	1338.7	655.3	593.5

a Total, as shown in the second column, is the wall time (in seconds) for completing the DFT job. SCF, Fock, ERI, and XC represent the average time (in seconds) to complete a single SCF step, Fock matrix construction, ERI, and XC calculation, respectively. ERI_MPI, XC_MPI, and Bcast_P are the average MPI communication times (in milliseconds) for reducing **J**, **K**, and **V**^*xc*^ matrices and broadcasting density matrix **P**.

For ubiquitin, which comprises 1,231 atoms and 11,577 basis functions in total, the time required for energy calculations decreases consistently as the number of GPUs is increased, indicating strong scaling performance. On 128 GPUs, the SCF calculations can be completed in less than five minutes.

**Figure 3:**
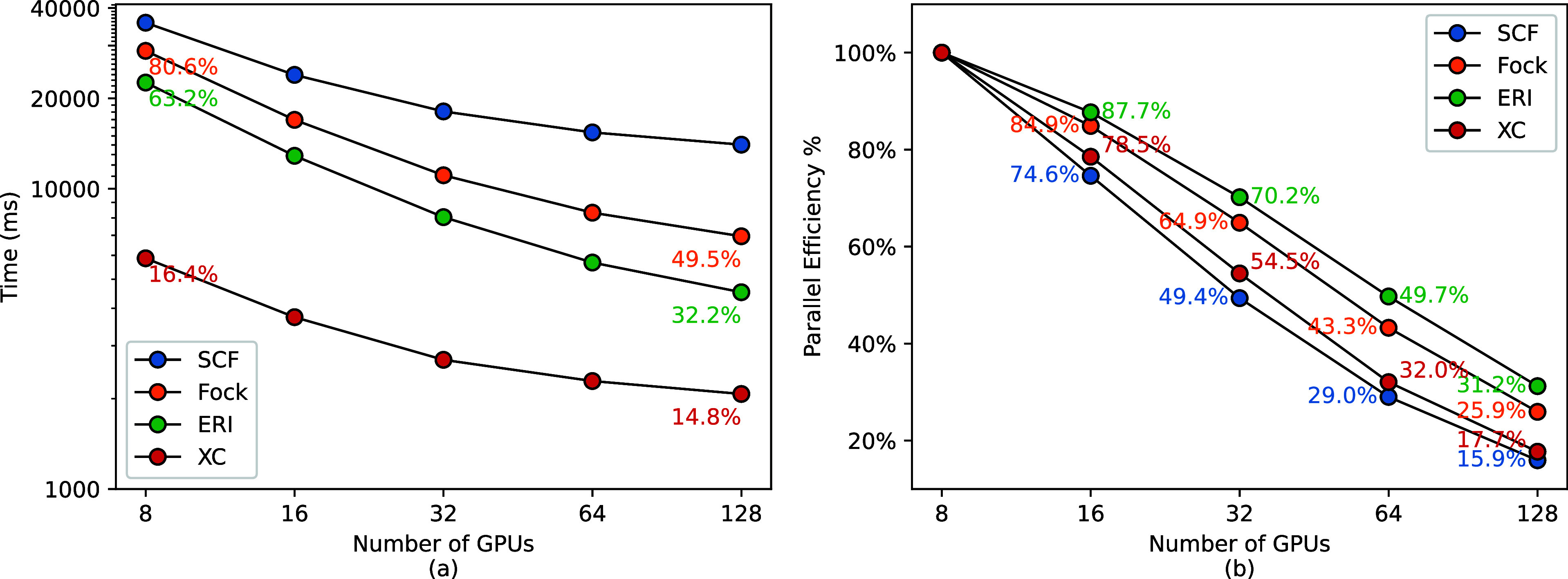
(a) Strong scaling performance of individual components within a single SCF step. Annotations indicate the contribution of each component to the overall SCF step. (b) Parallel efficiencies of each component with MPI communication costs included.

Figure 3 presents the scaling and parallel efficiency of the calculations. For SCF cycle and Fock matrix construction, 49.4 and 64.9% parallel efficiencies can be maintained at 32 GPUs, respectively. The performance gains become marginal when more computational nodes are added. On 128 GPUs, single-GPU calculations on rank 0, including Fock matrix diagonalization (3.4 s) and DIIS (2.5 s), account for 42% of the computational time in an SCF cycle. For ERI and XC calculations, 29.7 and 31% of the running time can be attributed to MPI communication cost, respectively. These analyses underscore the high efficiency of Accelerated DFT, exemplified by its ability to complete energy calculations for extremely large systems in a very short time. Nevertheless, the observed challenges in optimizing parallel efficiency in certain components of the SCF step highlight the need for further optimization strategies to enhance the overall scalability and efficiency of Accelerated DFT.

## Conclusions and Outlook

We present Accelerated DFT, a GPU-powered cloud-native code with the best performance in the presented benchmarks among selected computational chemistry codes. The high accuracy and efficiency of Accelerated DFT provide an opportunity to substantially expedite research across a wide spectrum of disciplines, from materials science to pharmaceutical development. The acceleration in computational capabilities enables the study of complex molecular systems, which previously required extensive computational resources and time, to be analyzed rapidly and with high precision.

Beyond traditional quantum chemistry research, Accelerated DFT will expedite AI model developments in the chemistry domain by generating high-quality data more efficiently. In this way, it will catalyze the overall development of new, faster development cycles in chemical research, which will open new frontiers in chemical space and in the discovery of novel molecules such as drugs beyond those available to standard DFT.

## Supporting Information Available

Additional benchmark results, speedup comparisons, and all structures and DFT input parameters used in the benchmarks (PDF)
