# Supplementary material for: Acceleration without Disruption: DFT Software as a Service
Source: J Chem Theory Comput. 2024 Dec 11;20(24):10838–51. doi: 10.1021/acs.jctc.4c00940 (PMC11673099; doi:10.1021/acs.jctc.4c00940)
Supplement: Supplementary file 1 — ct4c00940_si_001.pdf [file ct4c00940_si_001.pdf]

Supporting Information

Acceleration without Disruption: DFT Software as a Service

Fusong Ju,[†,§] Xinran Wei,[†,§] Lin Huang,[†,§] Andrew J. Jenkins,[‡,§] Leo Xia,[†] Jia Zhang,[†] Jianwei Zhu,[†] Han Yang,[¶] Bin Shao,[†] Peggy Dai,[†] David Williams-Young,[‡] Ashwin Mayya,[‡] Zahra Hooshmand,[‡] Alexandra Efimovskaya,[‡] Nathan A. Baker,[‡] Matthias Troyer,[‡] and Hongbin Liu[*,‡]

†*Microsoft Research AI for Science, Beijing, 100080, China*

‡*Microsoft Azure Quantum, Redmond, WA, 98052, USA*

¶*Microsoft Research AI for Science, Shanghai, 200232, China*

§*Contributed equally to this work*

E-mail: Hongbin.Liu@microsoft.com

Supporting Information Available

Figure S1 shows the speedup of Accelerated DFT over leading quantum chemistry software for M06-2X and ωB97x XC-functionals for the 329 molecule test set.

Figures S2-S3 shows the pairwise comparison of speedup of Accelerated DFT and leading quantum chemistry software for M06-2X and ωB97x XC-functionals for the 329 molecule test set.

Figures S4-S5 show a pairwise comparison of the compute time of each software for the full test set of molecules. The zero-interception linear regression fit is also plotted; a y=x line would indicate equal compute times for the software. Individual points are shaded on a scale from light to dark blue according to the number of basis functions used for that molecule. Accelerated DFT shows a consistently much shorter compute time than all other software.

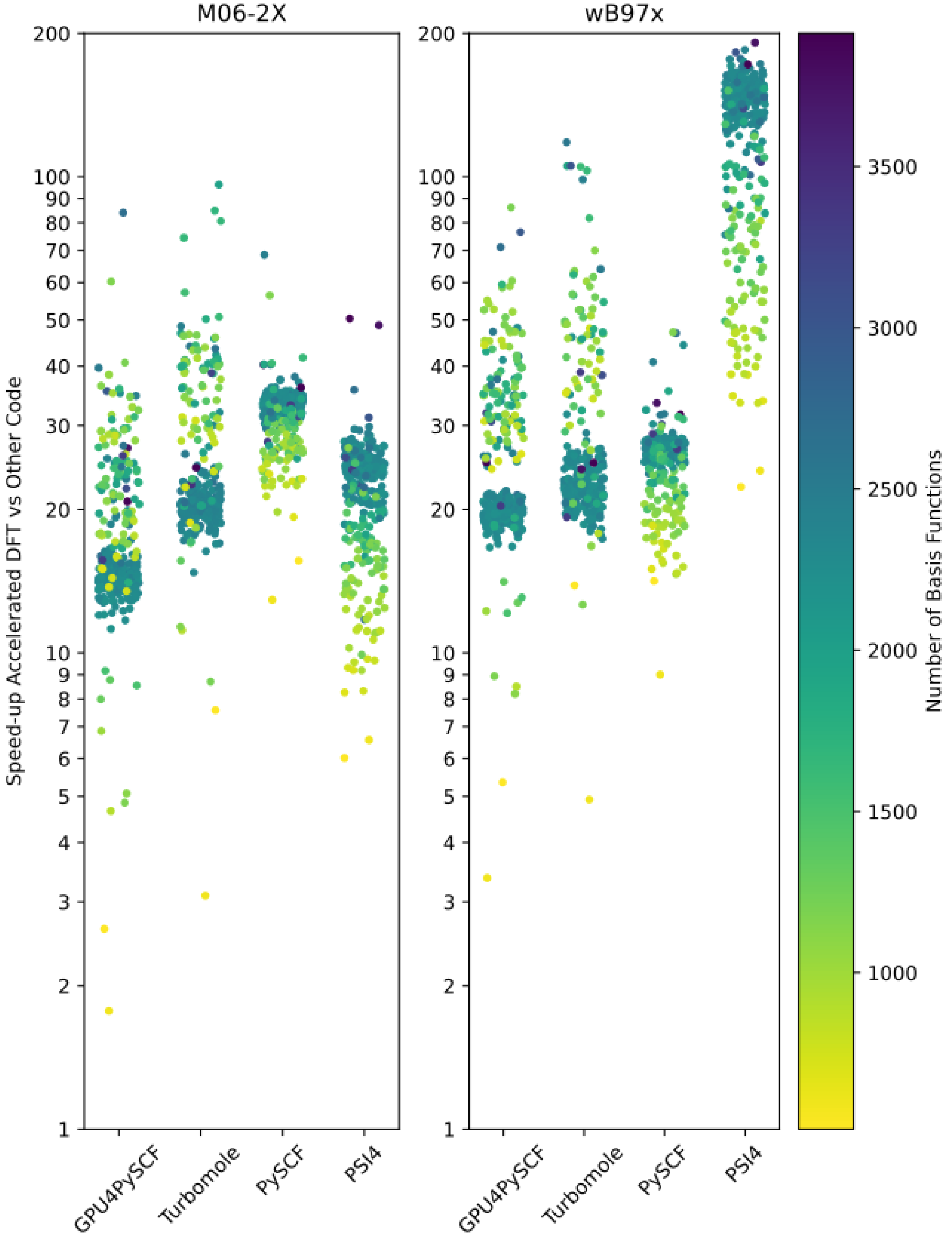

Figure S1: Speedup of Accelerated DFT over other software for the 329 molecule test set. The smallest speedup compared to to GPU4PySCF is 1.77 for the system water10 (30 atoms and 590 basis functions) and the largest speedup is 83.99 for the system revxx11135 (118 atoms and 2699 basis functions).

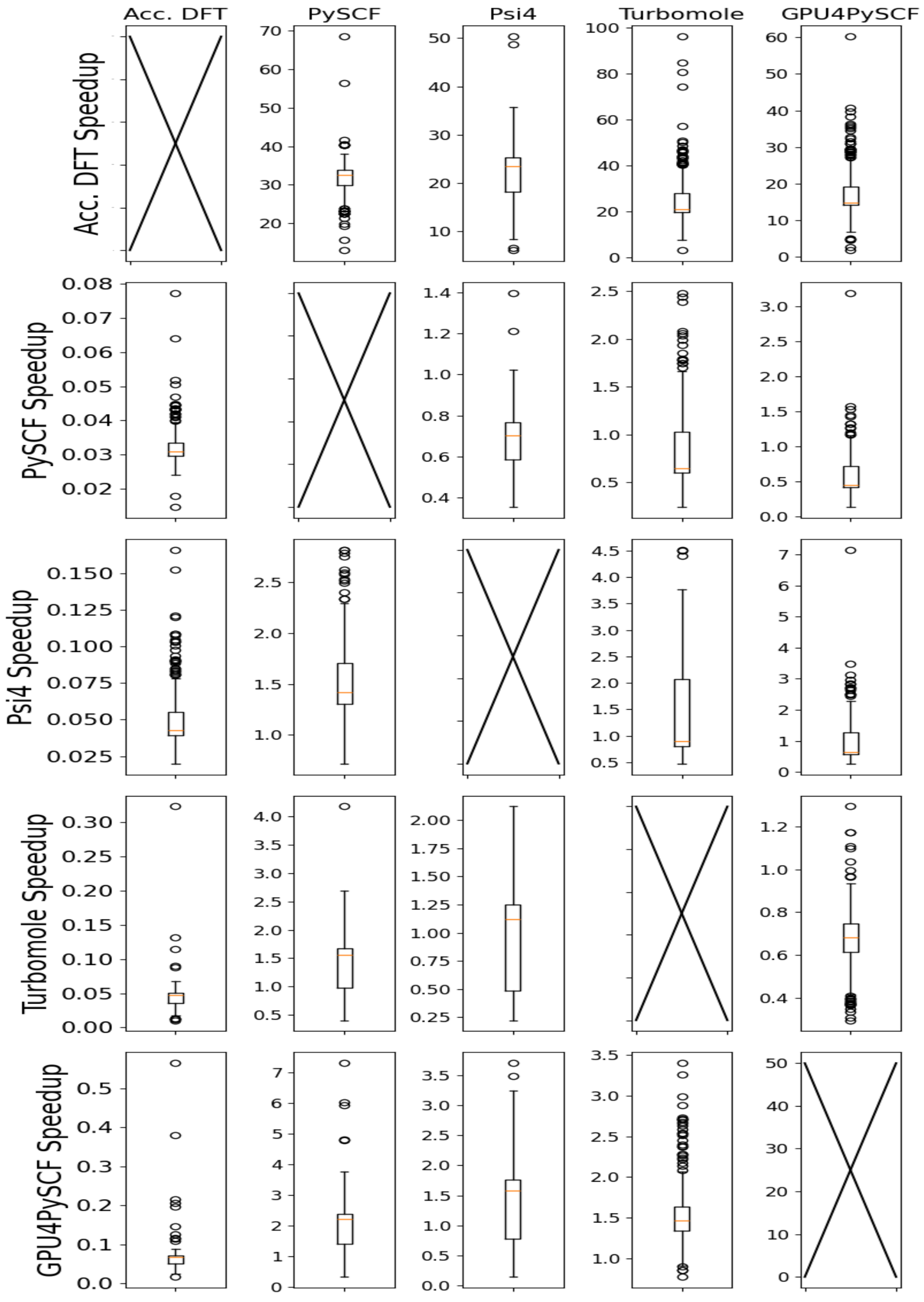

Figure S2: Boxplot of DFT calculations showing the speedup of Accelerated DFT over leading quantum chemistry software for M06-2X XC functional on the test set. The y-axis is adjusted for each pairwise comparison for visual purposes. The orange line inside each box indicates the median speedup, and the data points outside the whiskers represent the outliers. Accelerated DFT shows a consistent and significantly larger speedup over all other studied software.

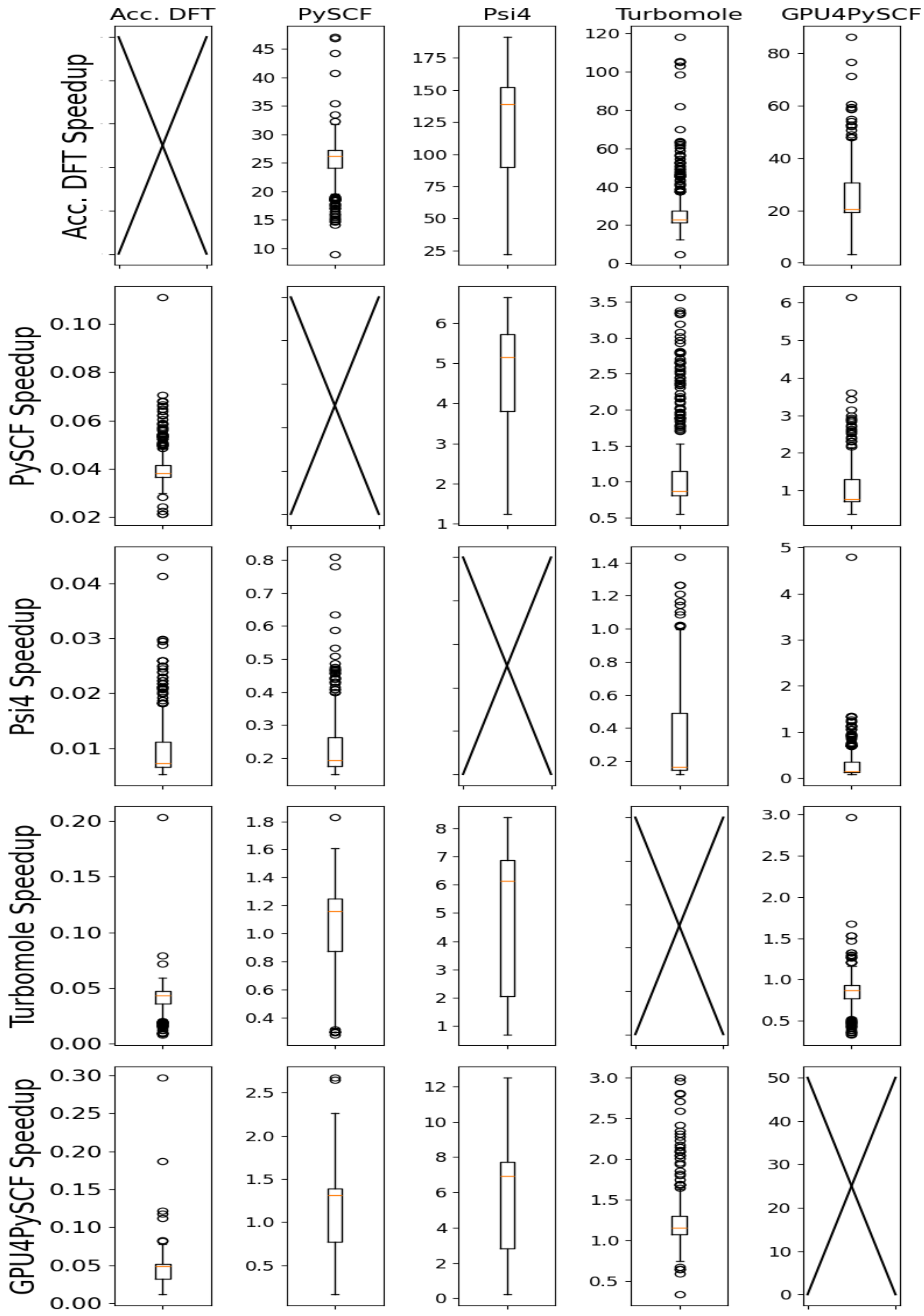

Figure S3: Boxplot of DFT calculations showing the speedup of Accelerated DFT over leading quantum chemistry software for ωB97X XC functional on the test set. The y-axis is adjusted for each pairwise comparison for visual purposes. The orange line inside each box indicates the median speedup, and the data points outside the whiskers represent the outliers. Accelerated DFT shows a consistent and significantly larger speedup over all other studied software.

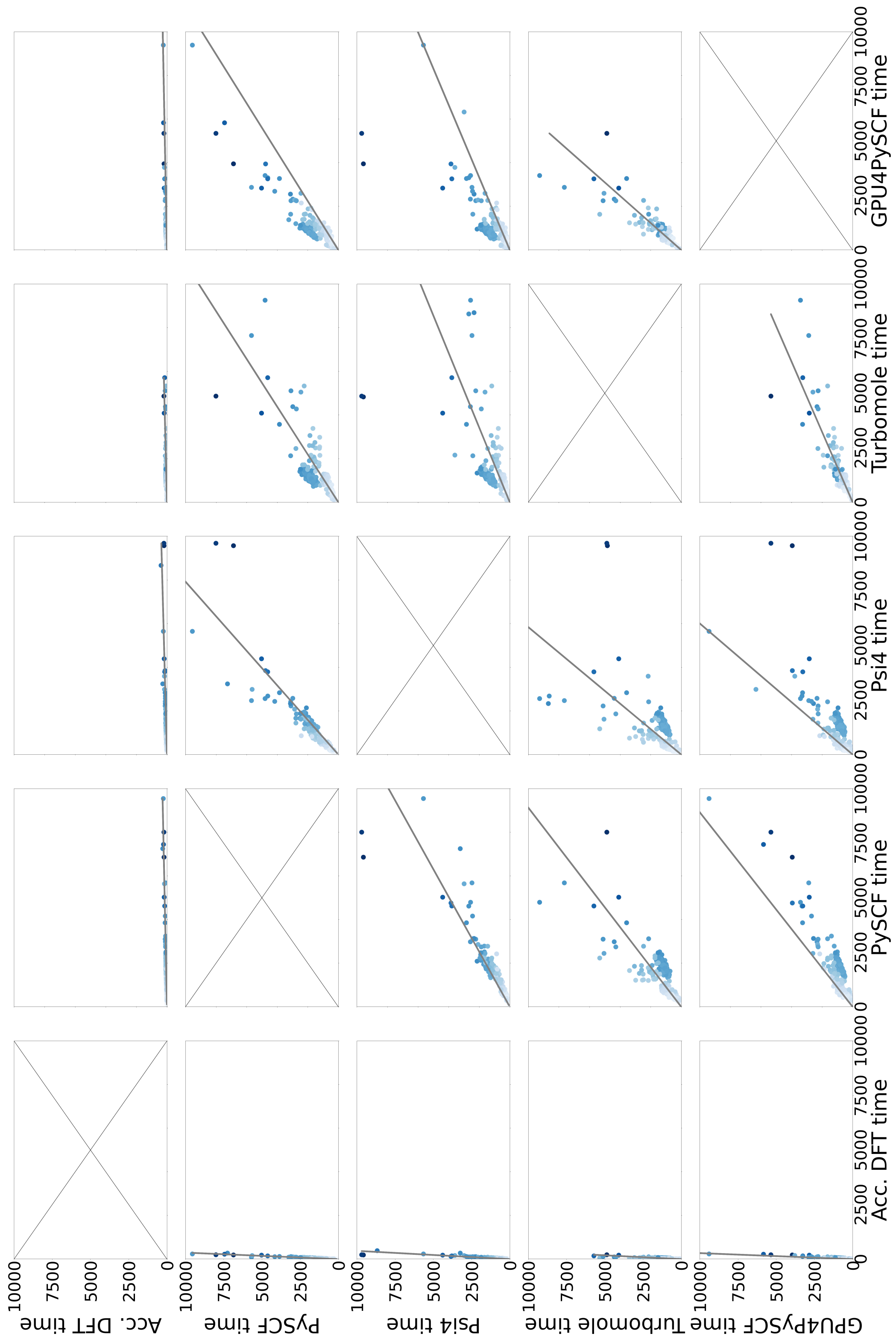

Figure S4: Parity plots of single point energy runtime between different DFT codes on the 329-molecule dataset using M06-2X. The solid line is the zero-interception linear regression fitting. Individual points are shaded on a scale from light to dark blue according to the size (number of basis functions) of that molecule

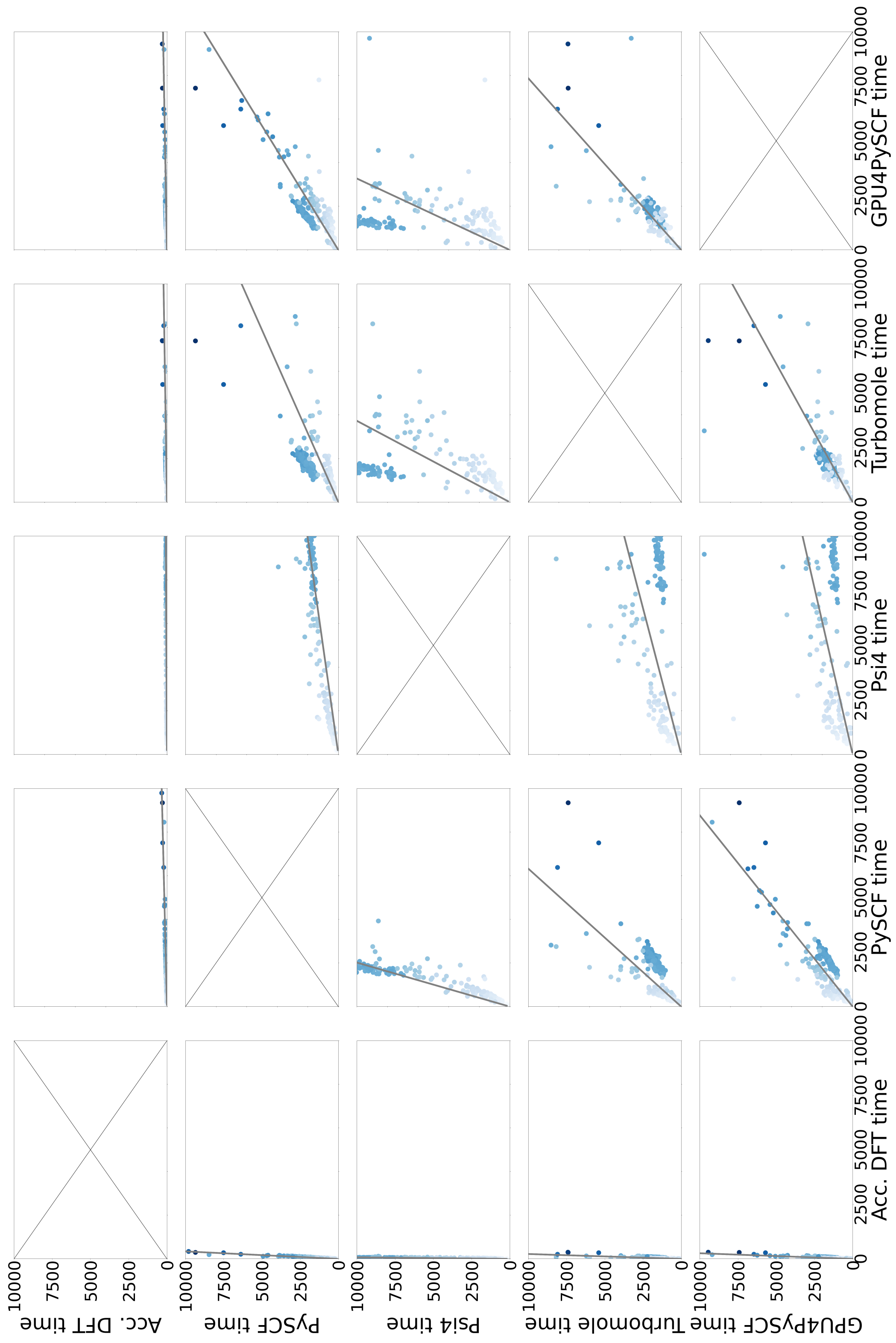

Figure S5: Parity plots of single point energy runtime between different DFT codes on the 329-molecule dataset using ωB97X. The solid line is the zero-interception linear regression fitting. Individual points are shaded on a scale from light to dark blue according to the size (number of basis functions) of that molecule

List S1 provides an input example of an Accelerated DFT job of single point energy calculation.

```
dft_input_params = {
    "tasks": [
    {
        "taskType": "spe",
        "molecule": { "charge": 0, "multiplicity": 1 },
        "scf": { "method": "rks", "maxSteps": 100, "convergeThreshold": 1e-8, "requireWaveFunction": True}
        "basisSet": { "name": "def2-svp" },
        "xcFunctional": { "name": "m06-2x", "gridLevel": 4 },
    }
    ]
}
```

Listing S1: Example of simple single point energy calculations input.

Support for PCM solvation models and Grimme-D3 dispersion[1,2] are included as additional keywords in the SCF field. Listing S2 shows an example of defining a geometry optimization in water solvent through IEFPCM and with dispersion correction.

```
dft_input_params = {
    "tasks": [
    {
        "taskType": "go",
        "molecule": { "charge": 0, "multiplicity": 1 },
        "scf": { "method": "rks", "maxSteps": 100, "convergeThreshold": 1e-8, "dispersion":"d3bj", "pcm": {"solverType": "IEFPCM", "solvent": "water"},"requireWaveFunction": True}
        "basisSet": { "name": "def2-svp" },
        "xcFunctional": { "name": "m06-2x", "gridLevel": 4 },
    }
    ]
```

```
}
```

Listing S2: Example of adding PCM solvation model and dispersion corrections to DFT calculations.

For GO and BOMD tasks, users can supplement with task-specific input parameters. Listing S3 shows how to adjust the convergence criteria for the geometry optimization, changing the energy threshold as well as the maximum and RMS values of the gradient and displacement.

```
    "tasks": [
      {
        "taskType": "go",
        "molecule": { "charge": 0, "multiplicity": 1 },
        "basisSet": { "name": "def2-svp"},
        "xcFunctional": { "name": "m06-2x", "gridLevel": 4 },
        "scf": { "method": "rks", "maxSteps": 100, "convergeThreshold": 1e-8 }
        "geometryOptimization": {"convergence_energy": 1e-6, "convergence_grms": 0.0003, "convergence_gmax": 0.00045, "convergence_drms": 0.0012, "convergence_dmax":0.0018 }
      }
    ]
```

Listing S3: Example of GO Input.

Listing S4 shows how to use the “molecularDynamics” field to set the time propagation, thermostat, timeStep in femtoseconds and temperature in the BOMD calculations. Currently, only the Berendsen thermostat[3] is implemented and supported in Accelerated DFT. The results of the calculations can be directly consumed and rendered in VMD.[4,5]

```
    "tasks": [
      {
        "taskType": "bomd",
        "molecule": { "charge": 0, "multiplicity": 1 },
```

```
            "basisSet": { "name": "def2-svp"},
            "xcFunctional": { "name": "m06-2x", "gridLevel": 4 },
            "scf": { "method": "rks", "maxSteps": 100, "convergeThreshold": 1e-8 },
            "molecularDynamics":{"steps": 100, "temperature": 300, "timeStep": 1, "thermostat": {"type": "berendsen", "timeSmoothingFactor": 0.05 } }
        }
    ]
```

Listing S4: Example BOMD Input.

In cases of GO and BOMD tasks, if no task-specific field is given, the default setting (as shown in Listing S3 and Listing S4) will be used.

If the requireWavefunction field has been set to True, the MOs will be stored in the Azure storage account and can be retrieved and loaded directly as a PySCF object with the snippet of code in Listing S5.

```
output = job.get_results()
mol, ks = create_scf_obj(output)
```

Listing S5: Example creating SCF and mol object in PySCF.

At this step, the “ks” object can be consumed as a normal PySCF KS object for various properties. An example is shown in Listing S6.

```
# Dipole Moment
dm = ks.make_rdm1(ks.mo_coeff, ks.mo_occ)
DipMom = ks.dip_moment(ks.mol, dm, unit='Debye', verbose=3)
# Molecular Electrostatic Potential
cubegen.mep(mol, 'file.cube', ks.make_rdm1())
# CHELPG and RESP atomic charges
q = chelpg_charges(ks)
p = resp_charges(ks)
# NMR chemical shifts
```

```
    nmr.RKS(ks).kernel()
    # Polarizability
    polarizability.rks.Polarizability(ks).polarizability()
```

Listing S6: Example of calculating dipole moment from PySCF DFT object.

If a Hessian 'fh' task was carried out in Accelerated DFT, the Hessian can also be loaded and used in the calculation of the vibrational frequencies and thermochemistry information as shown in Listing S7

```
    # Load Hessian from QcSchema output
    h = load_qcschema_hessian(output)

    # Compute Vibrational Frequencies
    freq = harmonic_analysis(mol,h)
    dump_normal_mode(mol,freq)

    # Compute Thermochemistry
    thermochem = thermo(ks,freq['freq_au'], 298.15)
```

Listing S7: Example of calculating vibrational frequenices and thermochemistry information in PySCF using the Accelerated DFT Hessian

This information can also be used to compute infrared spectra:

```
    # Load Hessian and prepare data
    ks_ir = prepare_ir(ks,output)
    # Compute IR spectrum
    infrared.rhf.kernel_dipderiv(ks_ir)
    ir_intensity = infrared.rhf.kernel_ir(ks_ir)
    # Plot the spectrum
    fig = ks_ir.plot_ir()[0]
    fig.savefig("ir_spectrum.png")
```

Listing S8: Example of calculating an infrared spectrum using the Accelerated DFT Hessian

The following additional information is available from github.com/microsoft/accelerated-dft:

1. The 329 molecular structures in xyz coordinates reported in the Benchmark section of the paper.
2. The input settings of running DFT calculations on the 329 molecular structures, including functionals, basis set, grid, and convergence criteria.
3. The total electronic energies and timings reported from the above mentioned Accelerated DFT calculations.

References

(1) Grimme, S.; Antony, J.; Ehrlich, S.; Krieg, H. A consistent and accurate ab initio parametrization of density functional dispersion correction (DFT-D) for the 94 elements H-Pu. The Journal of Chemical Physics **2010**, 132, 154104.

(2) Grimme, S.; Ehrlich, S.; Goerigk, L. Effect of the damping function in dispersion corrected density functional theory. Journal of Computational Chemistry **2011**, 32, 1456–1465.

(3) Berendsen, H. J. C.; Postma, J. P. M.; van Gunsteren, W. F.; DiNola, A.; Haak, J. R. Molecular Dynamics with Coupling to an External Bath. The Journal of Chemical Physics **1984**, 81, 3684–3690.

(4) Humphrey, W.; Dalke, A.; Schulten, K. VMD – Visual Molecular Dynamics. Journal of Molecular Graphics **1996**, 14, 33–38.

(5) Visual Molecular Dynamics. `http://www.ks.uiuc.edu/Research/vmd/`, Accessed: May 25, 2024.
